# Troubled times: Canadian social workers’ early adversities, mental health, and resilience during the COVID-19 pandemic

**DOI:** 10.1177/00208728221144380

**Published:** 2023-01-05

**Authors:** Ramona Alaggia, Carolyn O’Connor, Esme Fuller-Thomson, Keri West

**Affiliations:** University of Toronto, Canada

**Keywords:** Adversities, COVID-19, mental health, resilience, social workers, trauma informed approaches

## Abstract

Canadian social workers were surveyed about early adversities, mental health, and resilience. Bivariate analysis (*n* = 236) was conducted to understand relationships between predictor and outcome variables; and logistic regression analyses were conducted for depression, post-traumatic stress disorder, anxiety, and resilience. The impact of pandemic-related factors was also investigated. The results indicate that social workers are experiencing concerning levels of mental health issues, with significantly lower levels of resilience in younger social workers. A trauma and resilience informed approach to workplace policies and practices is urgently required to support social workers’ mental health needs.

## Introduction

Examining the health and well-being of social workers in Canada is essential for the development of equitable and supportive workplace practices. As such, we need a clearer understanding of social workers’ adversities, mental health, and resilience. This is required to capture a more complete picture of the state of social workers’ overall well-being, given that early research on social work professionals found significant histories of childhood adversity. In the context of the unprecedented strain of the global pandemic, this knowledge is of even greater importance.

Social workers occupy a vital role in society by supporting the optimal health, mental health, and well-being of individuals, families, and communities across the life course. They work with people to identify and aid in the remediation of psycho-social stressors, trauma, mental health challenges, and recovery from mental and physical illnesses. Many are at the frontlines engaging people with complex needs, who are facing poverty, oppression, and marginalization, alongside unprecedented hardships and complexities emerging in the face of a global pandemic. A great deal of social work is rooted in crisis work and trauma response. In addition, they work at the community level to eradicate structural barriers and disenfranchisement, as well as promote equity and social justice. As such, secondary trauma, compassion fatigue, and early departures from the field are common (Baird and Jenkins, 2003; Jenkins et al., 2011; Michalopoulos and Aparicio, 2012; Siebert, 2005; Travis et al., 2016).

Dating back to the 1990s, investigations focused on social workers’ histories to examine dysfunctions in their family of origin and motivations for entering social work (e.g. Deal, 1999; Elliott and Guy, 1993; Regehr et al., 2001; Rompf and Royse, 1994; Russel et al., 1993; Sellers and Hunter, 2005; Söderfeldt et al., 1995; Vincent, 1996). Since that time period, there has been a renewed interest in researching social workers, with the use of the adverse childhood experiences (ACEs) survey as a framework for understanding childhood adversities and their impact on social work professionals. Most of these studies focus on social work students, probing mental health issues, adversities, and connections to their choice of profession (Esaki and Larkin, 2013; Gilin and Kauffman, 2015; Horton et al., 2009; Newcomb et al., 2017; Olson and Royse, 2006; Palomino-Coila and Nunez-Palomino, 2020), although a few studies on actively practicing social workers and related helping professionals have been conducted more recently (Keesler, 2018; Stanley et al., 2021). Unfortunately, many studies define their target population in varying ways – ‘mental health practitioners’, ‘helping professionals’, ‘support workers’, while some exclusively focus on social workers. It is not always clear who has been sampled in terms of their professional affiliation. Furthermore, a systematic review of studies on trauma histories and impact on mental health practitioners noted that the vast majority are Western-based studies (Leung et al., 2022).

### Overview of research literature

It has been found that social workers report more childhood adversities than the general population, and also as compared with other professions (Black et al., 1993; Elliott and Guy, 1993; Keesler, 2018; Regehr et al., 2001; Rompf and Royse, 1994; Russel et al., 1993; Söderfeldt et al., 1995; Vincent, 1996). Historically, these studies found relatively high rates of psycho-social trauma, ACEs, and/or family dysfunction of social work students when compared with students enrolled in other programs (Black et al., 1993; Russel et al., 1993). Russel et al. (1993) found that almost three-quarters (73%) of Master of Social Work (MSW) students had experienced one or more problems that defined a dysfunctional family. In addition, as compared with students in other disciplines, social work students were significantly more likely to have a family member with a drug or alcohol abuse problem (50%); a family member who was a victim of a violent act (19%); and had themselves been sexually molested (31%; Russel et al., 1993). When compared with business students, MSW students had experienced a higher frequency of family trauma before the age of 21 years, including the death of a family member, alcohol or drug abuse, suicide, physical or mental illness, and physical, sexual, or emotional abuse (Black et al., 1993).

New developments in research have included using the groundbreaking work of Felitti et al., (1998) on childhood adversities and their health and mental health effects in adulthood. This team conducted a large survey in a US Health Maintenance Organization (HMO) to identify the prevalence of adverse events in early life and how these were associated with unhealthy behaviors (e.g. smoking, substance abuse), and long-term physical and mental health conditions (Felitti et al., 1998). They found that as the number of ACEs increased in the population studied, so did the risk of experiencing a range of health and mental health conditions in adulthood (Felitti et al., 1998). These childhood adversities include physical abuse, sexual abuse, parental addictions, parental mental illness, parental domestic violence, parental incarceration, and parental divorce. Serious mental health outcomes include depression, anxiety disorders, trauma, and suicidality (Felitti and Anda, 2014).

Studies with social work students have produced interesting results using the ACE framework. Gilin and Kauffman (2015) examined the ACE scores of social work graduate students and compared them with the results from the original ACE study of adult health clinic patients (Felitti et al., 1998). Several items had a considerably higher prevalence for the social work students than the clinic patients including parental separation or divorce (51% vs 23%), having an incarcerated household member (17% vs 5%), and emotional abuse (25% vs 11%). It was also noted that 27 percent of students had endorsed four or more ACE items compared with 13 percent of the original HMO study sample (Felitti et al., 1998; Gilin and Kauffman, 2015). A study by Thomas (2016) shows that 80 percent of MSW students experienced at least one ACE, with 42 percent endorsing four or more and 24 percent reporting six or more ACEs. Experiences most frequently identified by these students were parental divorce/separation (49%) and physical abuse (43%). These results, when compared with another sample of undergraduate university students, demonstrate that MSW students are 3.3 times more likely to report four or more ACEs (Thomas, 2016).

In a Peruvian study of social work students, 48 percent reported severe symptoms of stress, anxiety, or depression, some of which could be accounted for by college stressors, but there was an association with other significant factors from their personal histories (Palomino-Coila and Nunez-Palomino, 2020). Horton et al., (2009) also found that 34 percent of the participants in a social work program in an American university reported high levels of depressive symptoms, with 12 percent having a history of suicidal ideation.

Studies of social work professionals actively working in the field show similar findings. Keesler (2018) in his study found that about one-third (30%) of helping professionals working in disabilities services (direct support workers) had an ACE score of four or more. Those identifying as female and those who had been in their position less than 1 year had significantly higher ACE scores than males. The four most common ACE categories were parental divorce, a history of emotional abuse, parental mental illness, and parental substance abuse.

In contrast, Olson and Royse (2006) concluded that social workers’ histories were not necessarily characterized by more early life trauma and adversity when compared with a demographically similar comparison group of non-social workers, except in having greater familial mental illness (statistically significant) and emotional abuse (Olson and Royse, 2006).

Finally, in the largest systematic review focusing solely on trauma history among mental health practitioners, which included social workers, it was found that trauma history was associated with poorer mental health and negative changes in self-perception but was not associated with emotional exhaustion (i.e. burnout; Leung et al., 2022).

### Impact of the COVID-19 pandemic on social workers

Unique to this time in history, social workers have also been experiencing the effects of the COVID-19 pandemic. Regarding mental health, general population studies conducted across the world since the recent pandemic have shown increased levels of depression, anxiety, and post-traumatic stress disorder (PTSD) (Marić et al., 2022; Nochaiwong et al., 2021; Salari et al., 2020). Recent workforce trends indicate that women have felt the starkest negative impacts in terms of employment change, job loss, and having to leave the workforce during COVID-19 (Grekou and Lu, 2021; Moyser and Burlock, 2018). This is an important consideration given that the field of social work is comprised largely of women.

In a study examining American social workers in two waves of COVID-19 between 2020 and 2021, over a quarter (26.1%) of social workers met the diagnostic criteria for PTSD, which is higher than the national average. In addition, these study results showed that 64 percent of the 181 social workers surveyed reported burnout and 50 percent reported secondary trauma (Holmes et al., 2021).

Furthermore, a recently published paper on the impact of the COVID-19 pandemic on social workers in Canada identified substantial issues in the workplace context, including increased workload; clients presenting with more complex issues; job loss; job redeployment, challenges, and benefits transitioning to virtual methods of service delivery (Ashcroft et al., 2021). As well, themes in the personal context revealed respondents’ concerns for personal health and safety; personal caregiving responsibilities; taking early retirement; and concerns about negative impacts to their personal well-being (Ashcroft et al., 2021). However, some positive adaptations were also documented (Ashcroft et al., 2021).

### The role of resilience

Research on the prevalence of past adverse experiences among social workers and their related mental health outcomes are not always clearly identified in extant studies, and in particular the study of Canadian social workers’ resilience has been largely absent (Collins, 2007). As highlighted previously, there has also been an overreliance on studies focused on social work students rather than graduated, practicing social workers, as well as the use of small convenience samples.

Resilience theory underpins this study in examining predictors for higher or lower levels of resilience in social work professionals. This is a theory used to explain why some people can suffer adversities and have poorer mental health outcomes while others experiencing the same adversities show high resilience and good mental health outcomes (Leung et al., 2022; Rutter, 2012; Stanley et al., 2021). While adversities can be experienced at mild, moderate, or severe levels, mental health outcomes can vary because of specific individual, environmental, and contextual factors that offer protection and foster resilience (Ungar, 2013). To this end, we framed the current study using a holistic conceptualization of resilience as navigating through adversity, in which personal qualities, relationships, and community and cultural context support healthy adaptation and recovery over time. A holistic approach to resilience is based on social-ecological factors rather than focusing solely on individual (intra-personal) characteristics. As such, examining environmental factors attributed to resilience promotion is examined at all levels of the human ecology (ontogenic, interpersonal, mezzo, macro) (Alaggia & Donohue, 2018; Rutter, 2012). When faced with past adversities, past and current trauma, and job stressors, holistic resilience does not look only at the individual worker but rather encompasses the various environmental factors that support resilient responses (i.e. workplace conditions, availability of organizational supports, social supports).

Given the noted paucity of studies, there is a critical need for research on the prevalence of adversity, mental health outcomes, and levels of resilience among practicing social workers in Canada, particularly within the context of a global pandemic. The study questions were as follows:

1. What factors predict levels of resilience (high or low) among social workers in Canada?
2. What is the mental health status of social workers in terms of depression, anxiety, and PTSD in the context of the pandemic?
3. Do social workers facing more pandemic-related stressors demonstrate less resilience?

## Method

### Participants and procedure

A survey was distributed electronically through Qualtrics to social workers through various professional associations, like the Ontario Association of Social Workers, the Ontario College of Social Workers and Social Service Workers, the Canadian Association of Social Workers, the Canadian Association of Black Social Workers, and the Child Welfare League of Canada, and alumni listservs. The study was approved by the University of Toronto Health Sciences Research Ethics Board. The survey was launched on 9 January 2021 and was closed 28 February 2021. The sample in this study was restricted to the 236 respondents with complete data on all of the outcomes of interest and covariates used in the logistic regression analyses.

The survey probed for information about histories of adverse experiences in childhood based on the ACE survey, including: (1) form (physical abuse, sexual abuse, neglect, witnessing parental domestic violence, bullying, chronic illness, etc.), (2) frequency, (3) by whom the most serious incidents were perpetrated (parent, grandparent, sibling, neighbor, stranger, etc.) and at what age, (4) contact with child protection services/police, and (5) disclosure about the incidents (to a teacher, friend, spiritual leader, family member, etc.). The survey also inquired about adult mental health (depression, PTSD, anxiety), physical health and health behaviors, and protective factors/resilience (social support, spiritual coping, engagement with mental health services, etc.).

### Data collection tools

#### Measures

Standardized measures widely used in previous Canadian datasets were employed, allowing us to compare data from our non-random sample of social work volunteers to nationally representative samples. A demographics form was used along with depression, anxiety, PTSD, and resilience instruments, which are described below.

#### Outcome variables

##### Resilience

The sample’s resilience was measured using the 10-item version of the Connor–Davidson Resilience Scale (CD-RISC 10; Campbell-Sills and Stein, 2007). This self-report scale asks respondents to rate items on a 5-point Likert-type scale ranging from 0 (not true at all) to 4 (true nearly all the time). The total scores can range from 0 to 40, with a higher score indicating higher resilience. This variable was dichotomized at the 50th percentile (i.e. those with ‘high resilience’ were the upper 50th percentile and those with ‘low resilience’ at the lower 50th percentile) to run the bivariate analyses and logistic regressions.

##### Depression

The Center for Epidemiologic Studies Depression Scale Revised (CESDR-10) was used to measure levels of depression in the participants over the last week (Andresen et al., 1994). The CESDR-10 is a 10-item, self-report scale that asks about the frequency of depression symptoms (not at all or less than 1 day, 1–2 days, 3–4 days, or 5–7 days). Scores range from 0 to 30, and a cut-point of 10 or more was employed to indicate the presence of depression (Andresen et al., 1994). The cut-point separated the sample into two dichotomous categories: ‘Yes Depression’ for those who scored 10 or higher and ‘No Depression’ for those who scored less than 10.

##### Anxiety

Anxiety was measured using the 7-item Generalized Anxiety Disorders Scale (GAD-7; Spitzer et al., 2006). This self-report instrument assesses probable cases of GAD by asking about the presence of anxiety symptoms over the last 2 weeks (not at all, several days, more than half the days, or nearly every day). Scores range from 0 to 21, and a cut-point of 10 and above was used to indicate GAD (Spitzer et al., 2006). Again, the anxiety variable was dichotomized for the purposes of this article, with the cut-point demarcating ‘Yes Anxiety’ (10 or higher) and ‘No Anxiety’ (less than 10).

##### PTSD

To measure PTSD, we used the Primary Care PTSD Screen for *DSM-5* (PC-PTSD-5; Prins et al., 2016). The PC-PTSD-5 is a 5-item, self-report screen designed to assess how traumatic events have affected the participant over the past month, producing scores from 0 to 5 using ‘yes/no’ questions. Per recommendations by the measure’s authors, a cut-point score of 3 or more was deemed sensitive to identify probable PTSD in respondents (Prins et al., 2016). For the purposes of this article’s analyses, the PC-PTSD-5 was dichotomized into a binary variable, with scores of 3 or more categorized as ‘Yes PTSD’ and those who scored 2 or less as ‘No PTSD’.

#### Predictor variables

##### Demographics

A demographic questionnaire included a series of questions on personal characteristics such as age, racial/ethnic identity, gender, marital status, and income. In addition, information on each respondent’s professional history was collected to identify their educational degree or diploma, number of years spent working in the field, and area of specialization (e.g. child welfare, addictions, gerontology, school social work).

##### Adverse childhood experiences

Five types of adverse exposures occurring before the age of 18 were included in the analyses: Physical abuse, sexual abuse, parental problems, neglect, and feeling loved. Participants were coded as having been exposed to physical abuse if they responded that an adult had at least once kicked, bit, punched, choked, burned, or physically attacked them; slapped them on the face, head or ears, or hit them with something hard to hurt them; or pushed, grabbed, shoved, or threw something at them to hurt them. Participants were coded as having been exposed to sexual abuse if they responded that an adult had ever forced them or attempted to force them into any unwanted sexual activity, by threatening them, holding them down or hurting them in some way; or touched them against their will in any sexual way. Participants were coded as having experienced parental problems if they responded in the affirmative to any of the following: their parent or guardian had suffered from a mental or psychiatric illness or had had a breakdown; their parent or guardian drank or used drugs so often that it caused problems for the family; their parents divorced or separated; or they experienced the death or serious illness of their parent or primary caregiver. Participants were coded as having experienced neglect if they indicated both that their basic needs were unmet more than 10 times, and that they had no one to take them to the doctor when needed, at least sometimes. Those who responded that they sometimes, rarely, or never felt loved were coded as not having felt loved, whereas those who responded that they often or very often felt loved were coded as having felt loved.

##### COVID adversity index

A 9-item COVID adversity index was developed by the authors with items combined from a Statistics Canada survey related to COVID-19 for use in the logistic regression analyses to determine the stressors experienced by participants during the pandemic and their association with the outcome variables. Items drawn from the Statistics Canada Canadian Perspective Survey Series 1: Impacts of COVID-19 (Statistics Canada, Centre for Labour Market Information, 2020) included whether the respondent had concerns about their own health (not at all or somewhat/very much or extremely), household members’ health (not at all or somewhat/very much or extremely), family stress from confinement (not at all or somewhat/very much or extremely), and violence in the home (not at all or somewhat/very much or extremely). Questions added by the investigators asked about job loss (yes/no), increase in caregiving responsibilities (yes/no), stress associated with work–life balance (not at all or somewhat/very much or extremely), whether the participant had tested positive for COVID-19 (yes/no), and concerns about household members’ mental health (not at all or somewhat/very much or extremely). The index was scored from 0 to 9, with 0 indicating that the respondent endorsed none of the COVID-related adversities and 9 indicating that they endorsed all COVID-related adversities.

##### Analytic procedures

Several analytic techniques were employed to develop a profile of social workers in Canada and compare the results with those found in national datasets and global norms. Once the dataset was cleaned, descriptive statistics (means and standard deviations) were calculated for continuous variables such as age and number of ACEs endorsed. The majority of the remaining variables were categorical and as such, frequencies were calculated to determine the occurrence of each measure within the sample and chi-square statistics were performed. As previously mentioned, three of the four outcome measures were converted to binary variables by their respective cut-point scores to split participants into two groups: those who are currently experiencing the condition and those who are not. The fourth variable, resilience, was also dichotomized but at the 50th percentile mark, categorizing participants as having ‘high’ or ‘low’ resilience. This process allowed for the analysis of bivariate correlations and binary logistic regression models.

Bivariate analyses were conducted to understand the relationship between each of the predictor and outcome variables of interest, including whether any significant associations are present. Binary logistic regression analyses were conducted for all four outcome variables to determine the associated correlates with predictive value. Four sets of logistic regression analyses were undertaken regressing PTSD, depression, anxiety, and resilience on a similar set of predictors to determine which variables are associated with each outcome of interest.

As a point of reference, we used Chen et al. (2010) thresholds for interpretation of effect size for logistic regression: for odds less than 1, 0.60, 0.29, and 0.15 indicate small, medium, and large effects, respectively; and for odds greater than 1, 1.68, 3.47, and 6.71 indicate small, medium, and large effects, respectively. With respect to the interpretation of *p*-values, values around 0.05 show weak evidence against the null hypothesis, values less than 0.01 show strong evidence against the null hypothesis, and values less than 0.001 show very strong evidence against the null hypothesis.

## Results

The total sample for this study consisted of 236 social workers and social service workers. The participants’ age ranged from 20 to 91 (*M* = 42.98, *SD* = 14.16) for the 189 respondents who reported their age; an additional 45 respondents were classified as ‘missing’ age data. As indicated in Table 1, the majority of participants were female (84.3%), White (85.6%), and in married or common law relationships (69.5%). Just over half of the sample had children (53.4%). Socioeconomic status was measured by annual household and personal income, with 59.3 percent of participants in a household earning $100,000 or more and 41.1 percent earning a personal income between $50,000 and $79,999. Most participants’ highest degree earned was an MSW (89.8%). Other descriptive findings not reported in the tables include the following: On average, participants had been working in the field for 15.20 years (*SD* = 11.67). Among areas of career specialization, adult mental health (57.6%), child and family (32.6%), medical/physical health (19.1%), older adults/gerontology (13.6%), and domestic violence/violence against women (13.1%) were selected most often.

**Table 1.** Sociodemographic and mental health characteristics of a sample of Canadian social workers, gathered in January/February 2021 (*n* = 236).

Characteristics	*n*	%
Gender		
Male	27	11.4
Female	199	84.3
Other	10	4.2
Race/ethnicity		
White	202	85.6
Visible minority	34	14.4
Age		
Under 35 years	69	29.2
35–54 years	74	31.4
55+ years	46	19.5
Missing	47	19.9
Marital status		
Never married	48	20.3
Formerly married divorced, separated, widowed)	24	10.2
Married or common law	164	69.5
Children		
Yes	126	53.4
No	109	46.2
Household income		
Less than $100,000	96	40.7
$100,000+	140	59.3
Personal income		
Less than $50,000	46	19.5
$50,000–$79,999	97	41.1
$80,000+	93	39.4
Highest education level completed		
Social Service Worker	4	1.7
Bachelor of Social Work	11	4.7
Master of Social Work	212	89.8
PhD in Social Work	9	3.8
PC-PTSD-5		
Yes	49	20.8
No	187	79.2
CESDR-10		
Yes	96	40.7
No	140	59.3
GAD-7		
Yes	37	15.7
No	199	84.3
ACE frequencies		
0	46	19.5
1	69	29.2
2	62	26.3
3	35	14.8
4	13	5.5
5	11	4.7

PC-PTSD-5: Primary Care PTSD Screen for *DSM*-5; CESDR-10: Center for Epidemiologic Studies Depression Scale Revised; GAD-7: 7-item Generalized Anxiety Disorders Scale; ACEs: adverse childhood experiences.

### Descriptive statistics

Tables 1 and 2 describe the sample characteristics and descriptive statistics for the following mental health variables: depression, PTSD, and anxiety. Two in every five respondents (40.7%) scored above the cut-off for depression on the CESDR-10, about one-fifth (20.8%) of the sample scored above the cut-off on the PC-PTSD-5, and 15.7 percent scored at or above the cut-point for anxiety on the GAD-7. ACE frequencies ranged from 0 to 5 (no participant endorsed more than 5 ACEs) with an overall mean of 1.72 ACEs (*SD* = 1.33).

**Table 2.** Sociodemographic characteristics of a sample of Canadian social workers by CD-RISC, PC-PTSD-5, CESDR-10, and GAD-7. Data were gathered in January/February 2021 (*n* = 236).

Variable	*n*	CD-RISC 10 (high resilience) *n* = 121 (%)	*p*-value (χ^2^)	PC-PTSD-5 *n* = 49 (%)	*p*-value (χ^2^)	CESDR-10 *n* = 96 (%)	*p*-value (χ^2^)	GAD-7 *n* = 37 (%)	*p*-value (χ^2^)
Gender									
Male	27	66.7	0.235 (2.895)	0.00	0.016* (8.263)	37.0	0.424 (1.714)	11.1	0.372 (1.979)
Female	199	49.2		23.1		40.2		15.6	
Other	10	50.0		30.0		60.0		30.0	
Race/ethnicity									
White	202	50.0	0.341 (0.907)	19.3	0.179 (1.806)	39.6	0.413 (0.670)	15.8	0.866 (0.028)
Visible minority	34	58.8		29.4		47.1		14.7	
Age									
Under 35 years	69	37.7	0.042* (8.190)	27.5	0.102 (6.216)	47.8	0.139 (5.488)	–	–
35–54 years	74	54.1		24.3		33.8		–	–
55+ years	46	63.0		10.9		32.6		–	–
Missing	47	55.3		14.9		48.9		–	–
Marital status									
Never married	48	(58.3)	0.108 (4.460)	18.8	0.927 (0.152)	43.8	0.873 (0.271)	20.8	0.374 (1.968)
Formerly married	24	(66.7)		20.8		41.7		8.3	
Married or common law	164	(47.0)		21.3		39.6		15.2	
Household income									
Less than $100,000	96	(51.0)	0.953 (0.003)	19.8	0.761 (0.093)	43.8	0.426 (0.633)	17.7	0.477 (0.505)
$100,000+	140	(51.4)		21.4		38.6		14.3	
Personal income									
Less than $50,000	46	(37.0)	0.006** (10.210)	23.9	0.546 (1.210)	54.3	0.107 (4.474)	23.9	0.076 (5.143)
$50,000–$79,999	97	(46.4)		22.7		38.1		17.5	
$80,000+	93	(63.4)		17.2		36.6		9.7	
Number of ACEs									
0–2	177	88	0.408 (0.684)	19.2	0.308 (1.039)	39.0	0.359 (0.843)	16.9	0.352 (0.865)
3–5	59	33		25.4		45.8		11.9	

CD-RISC: Connor–Davidson Resilience Scale; PC-PTSD-5: Primary Care PTSD Screen for *DSM*-5; CESDR: Center for Epidemiologic Studies Depression Scale Revised; GAD-7: 7-item Generalized Anxiety Disorders Scale. ACEs: adverse childhood experiences.

* Significant at *p* < 0.05; **significant at *p* < 0.01; ***significant at *p* < 0.001.

### Bivariate analysis

Bivariate analyses were performed to determine any significant associations between our predictor and outcome variables (see Table 2). Bivariate analyses showed strong evidence (as defined by *p* < 0.01) of a positive correlation between personal income and resilience (*p* = 0.006,χ^2^ = 10.210, df = 2), such that those who make less than $50,000 per year were less likely to be resilient. Weak evidence (as defined by a *p* < 0.05) was found for a positive correlation between age and resilience (*p* = 0.042, χ^2^ = 8.190, df = 3), such that those under age 35 years were less likely to be resilient. No other significant bivariate differences were found with regard to resilience. Moderate evidence of a positive, significant association was also found between gender and PTSD (*p* = 0.016, χ^2^ = 8.263, df = 2), with participants identifying as women (*n* = 199, 23.1% with PTSD) or as a gender other than a man or woman (*n* = 10, 30% with PTSD) as more likely to have PTSD than those identifying as men (*n* = 27, 0% with PTSD). We did not find evidence of other significant bivariate relationships for either depression or anxiety.

### Logistic regression

Binary logistic regression analyses were conducted for all four outcome variables to determine the independent association between factors and each outcome, while keeping other covariates constant. Reference categories for each predictor variable are noted in the tables. To aid the reader in interpreting the size of the odds ratio (OR), it is helpful to note that Chen et al. (2010) propose that an OR equal to 1.68 is equivalent to a small effect size, 3.47 is equivalent to a medium effect size, and 6.71 is equivalent to a large effect size (Table 3).

**Table 3.** Prevalence of positive responses to questions contained in the COVID adversity index among a sample of social workers (*n* = 236) gathered in January/February 2021 and a nationally representative sample of Canadians aged 15 years and over (*n* = 4600) gathered in March/April 2020.

Item	Social worker participants (*n* = 236) (%)	95% CI	Statistics Canada participants (*n* = 4600) (%)	95% CI	Mean difference
Concerned about my own health^a^	23.7	18.3–29.2	36.4	34.4–38.5	
Concerned household members’ health^a^	36.4	30.3–42.6	54.2	52.8–57.5	
Concerned about family stress from confinement^a^	43.6	37.3–50.0	32.4	30.1–34.8	
Concerned about violence in the home^a^	1.3	0.0–2.7	8.1	6.9–9.5	
Lost job due to COVID	3.8	1.4–6.3			
Caregiving increased very much or extremely	24.6	19.1–30.1			
Concerned about household members’ mental health^a^	38.1	31.9–44.3			
Tested positive for COVID	0.8	0.0–2.0			
Concerned about work–life balance^a^	43.2	36.9–49.5			

*Source*: Findlay et al. (2020).

CI: confidence interval.

a Percentages represent those who selected ‘very concerned’ or ‘extremely concerned’ in response to each item.

Table 4 shows the binary logistic regression model with resilience as the outcome variable. Of note, the personal income under $50,000 category showed moderate evidence of an association, with a 65 percent reduced likelihood of being resilient (OR = 0.35, 95% CI 0.15–0.84, *p* = 0.02). Those with a personal income level of $50,000–$79,999 had 47 percent lower odds of being resilient (OR = 0.53, 95% CI 0.27–1.03) than those with a personal income of $80,000 or higher, however the *p*-value of 0.06 indicates the association failed to reach statistical significance. The Nagelkerke *R*^2^ suggests that the model explains about 15.1 percent of the variance in resilience (see Table 4).

**Table 4.** Multivariate logistic regression of CD-RISC by sociodemographic characteristics, early adversities, and COVID adversity index in a sample of social workers gathered in January/February 2021 (*n* = 236).

Predictors	Nagelkerke *R*^2^ = 0.15	
	OR	95% CI
Number of ACEs	0.91	0.72–1.14
Race/ethnicity		
White	1.00	Reference
Visible minority	1.35	0.60–3.06
Age		
Under 35 years	0.40	0.16–1.04
35–54 years	0.89	0.38–2.09
55+ years	1.00	Reference
Missing	0.70	0.28–1.77
Marital status		
Never married	1.00	Reference
Formerly married	0.75	0.22–2.51
Married or common law	0.47	0.20–1.10
Household income		
Less than $100,000	1.19	0.58–2.45
$100,000+	1.00	Reference
Personal income		
Less than $50,000	0.35*	0.15–0.84
$50,000–$79,999	0.53	0.27–1.03
$80,000+	1.00	Reference
Gender		
Male	1.00	Reference
Female	0.61	0.25–1.51
Other	0.68	0.13–3.63
COVID adversity index	0.86	0.73–1.02

CD-RISC: Connor–Davidson Resilience Scale; OR: odds ratio; CI: confidence interval; ACEs: adverse childhood experiences.

* Significant at *p* < 0.05; **significant at *p* < 0.01; ***significant at *p* < 0.001.

The next regression model, shown in Table 5, used PTSD as the outcome variable. Gender was not included in this analysis as the model became unstable when gender was included, due to small cell sizes of PTSD outcomes among men. There was weak evidence of an association between PTSD and the COVID adversity index (OR = 1.27, 95% CI 1.04–1.55, *p* = 0.02). Each additional COVID-related adversity was associated with a 27 percent higher risk of having PTSD. Our analyses provide weak evidence suggesting that respondents under aged 35 years had almost four times the odds of PTSD compared with those aged 55 years and older (OR = 3.76, 95% CI 1.07–13.22, *p* = 0.04). Although each additional ACE increased the odds of having PTSD by 28 percent (OR = 1.28, 95% CI 0.98–1.67, *p* = 0.07), this association failed to reach statistical significance at the 0.05 level. This model explains approximately 13.3 percent of the variance for PTSD (Nagelkerke *R*^2^).

**Table 5.** Multivariate logistic regression of PC-PTSD-5 by sociodemographic characteristics, early adversities, and COVID adversity index in a sample of social workers gathered in January/February 2021 (*n* = 236).

Predictors	Nagelkerke *R*^2^ = 0.13	
	OR	95% CI
Number of ACEs	1.28	0.98–1.67
Race/ethnicity		
White	1.00	Reference
Visible minority	1.56	0.63–3.85
Age		
Under 35	3.76*	1.07–13.22
35–54 years	2.57	0.81–8.17
55+ years	1.00	Reference
Missing	1.52	0.41–5.68
Marital status		
Never married	1.00	Reference
Formerly married	1.41	0.31–6.31
Married or common law	1.25	0.44–3.56
Household income		
Less than $100,000	0.74	0.31–1.78
$100,000+	1.00	Reference
Personal income		
Less than $50,000 (1)	1.42	0.50–4.01
$50,000–$79,999 (2)	1.20	0.53–2.71
$80,000+	1.00	Reference
Gender^a^		
Male	–	–
Female	–	–
Other	–	–
COVID adversity index	1.27*	1.04–1.55

PC-PTSD-5: Primary Care PTSD Screen for *DSM*-5; OR: odds ratio; CI: confidence interval; ACEs: adverse childhood experiences.

a Gender removed for this analysis.

* Significant at *p* < 0.05; **significant at *p* < 0.01; ***significant at *p* < 0.001.

Table 6 shows the multivariate logistic regression model for depression. There was very strong evidence (as defined by *p* < 0.001) for an association between the COVID adversity index and the depression outcome variable (OR = 1.53, 95% CI 1.28–1.83). With each added COVID-related adversity, there is 53 percent higher odds of depression. Our analyses also provided weak evidence that those with a personal income under $50,000 had double the odds of being depressed when compared with those whose incomes were $80,000 or higher (OR = 2.18, 95% CI 0.90–5.30, *p* = 0.09). The Nagelkerke *R*^2^ indicates that the overall model explains about 20.2 percent of the variation in depression outcomes.

**Table 6.** Multivariate logistic regression for the CESDR-10 by sociodemographic characteristics, early adversities, and COVID adversity index in a sample of social workers gathered in January/February 2021 (*n* = 236).

Predictors	Nagelkerke *R*^2^ = 0.20	
	OR	95% CI
Number of ACEs^a^	1.17	0.92–1.48
Race/ethnicity		
White	1.00	Reference
Visible minority	1.03	0.44–2.41
Age		
Under 35 years	1.55	0.58–4.15
35–54 years	0.90	0.37–2.24
55+ years	1.00	Reference
Missing	2.02	0.77–5.26
Marital status		
Never married	1.00	Reference
Formerly married	0.91	0.27–3.08
Married or common law	0.73	0.31–1.73
Household income		
Less than $100,000	0.82	0.39–1.70
$100,000+	1.00	Reference
Personal income		
Less than $50,000	2.18	0.90–5.30
$50,000–$79,999	0.99	0.50–2.01
$80,000 +	1.00	Reference
Gender		
Male	1.00	Reference
Female	0.85	0.34–2.11
Other	1.27	0.25–6.60
COVID adversity index	1.53***	1.28–1.83

CESDR-10: Center for Epidemiologic Studies Depression Scale Revised; OR: odds ratio; CI: confidence interval; ACEs: adverse childhood experiences.

* Significant at *p* < 0.05; **significant at *p* < 0.01; ***significant at *p* < 0.001.

The final logistic regression model was performed with anxiety as the outcome variable (see Table 7). The age variable was split dichotomously in this model (under 45 and 45 or older) as using the previous three level age split (under 35, 35–54, and 55+ years) resulted in unstable estimates. Similar to the results above, there was very strong evidence (as indicated by *p* < 0.001) that the COVID adversity index variable was associated with anxiety (OR = 1.59, 95% CI 1.25–2.03). This model demonstrated the highest associated risk when compared with the previous regression models, as each COVID adversity increased the likelihood of anxiety by 59 percent. The final model explains 19.8 percent of the variance in anxiety according to the Nagelkerke pseudo *R*^2^ statistics. It should be noted that in Block 2, before the COVID adversity index variable was added, the explained variance was 9.3 percent; therefore, the COVID index alone explained an additional 10.5 percent of the variation in anxiety outcomes, more than all other variables combined.

**Table 7.** Multivariate logistic regression for GAD-7 by sociodemographic characteristics, early adversities, and COVID adversity index in a sample of social workers gathered in January/February 2021 (*n* = 236).

Predictors	Nagelkerke *R*^2^ = 0.20	
	OR	95% CI
Number of ACEs	1.05	0.76–1.45
Race/ethnicity		
White	1.00	Reference
Visible minority	0.69	0.22–2.19
Age^a^		
Under 45 years	1.87	0.55–6.35
45+ years	1.00	Reference
Missing	2.44	0.67–8.90
Marital status		
Never married	1.00	Reference
Formerly married	0.43	0.07–2.67
Married or common law	0.48	0.16–1.44
Household income		
Less than $100,000	0.68	0.25–1.81
$100,000+	1.00	Reference
Personal income		
Less than $50,000	2.77	0.84–9.20
$50,000–$79,999	1.66	0.59–4.69
$80,000+	1.00	Reference
Gender		
Male	1.00	Reference
Female (1)	0.82	0.21–3.18
Other (2)	1.83	0.24–13.75
COVID adversity index	1.59***	1.25–2.03

GAD-7: 7-item Generalized Anxiety Disorders Scale; OR: odds ratio; CI: confidence interval; ACEs: adverse childhood experiences.

a Age variable split dichotomously for this analysis.

* Significant at *p* < 0.05; **significant at *p* < 0.01; ***significant at *p* < 0.001.

## Discussion

Results of this investigation contribute to a better understanding of social workers’ adversities, mental health, and resilience during the COVID-19 pandemic. Findings in the current study were drawn from a cross-sectional convenience sample (*n* = 236) with predominantly White women from Ontario, Canada, with an MSW, working as social workers with clients across a wide age range in mental health, health, and related psycho-social issues. Most respondents worked in adult mental health, and child and family specializations. The majority were married or in common law unions, and half of respondents had children under 18 years of age. These profiles are remarkably similar to a recent study of social workers in Canada and their characteristics (Ashcroft et al., 2021) and in global studies (Keesler, 2018; Stanley et al., 2021).

Most noteworthy of these study findings is the observed relationship between COVID-19 stressors and social workers’ mental health. Using the COVID-19 adversity index, we found that this was the strongest factor explaining depression, PTSD, and anxiety. It is not surprising that those experiencing the greatest number of COVID-related stressors succumbed to mental health problems at a higher rate compared with those who were not as impacted by pandemic-related hardship.

Findings from this survey suggest that income and age had some bearing on resilience, as older age and higher income were associated with higher resilience scores. These findings are comparable to those found in an Indian study that found older, more established social workers had higher resilience scores (Stanley et al., 2021). We hypothesize that more stressors are present in the earlier stages of careers, marked by less experience and lack of control over job conditions, while simultaneously earning a lower wage, an environmental factor that adds to this stress. Early in a social worker’s career trajectory, job insecurity and instability are common factors, especially within a neo-liberal environment that promotes contractual, and therefore, precarious, work conditions (Pentaraki, 2017; Söderfeldt et al., 1995). As well, it has been established that women working in Canada are more marginalized than men in the workforce as they are often in life circumstances with childcare and domestic responsibilities that drive them to part-time and contractual paid employment arrangements, with recent trends indicating that women have felt the most negative employment change and job loss during COVID-19 (Grekou and Lu, 2021; Moyser and Burlock, 2018).

In terms of mental health, of great concern was that depression was reported by 40.7 percent of social work respondents. When compared with the Canadian General Social Survey (GSS) indicating that 11.7 percent of employed respondents experienced major depressive episodes between 2010 and 2016 (Dobson et al., 2020), the current study sample has almost four-fold higher levels of depression. This is also substantially higher when compared with the general population, with a lifetime prevalence for depression ranging from 7 percent to 21 percent (Bromet et al., 2011; Vilagut et al., 2016). However, caution should be exercised in interpretation as most of the previous discussion draws from literature conducted in the pre-pandemic era and, therefore, may not be directly comparable to our data gathered during 2021.

Most importantly, the current survey results indicated substantially higher levels of depression than other health care professionals working in COVID-19 related conditions. For example, recent systematic reviews found a prevalence rate of 24.3 percent for depression among health care workers during COVID-19 (Salari et al., 2020) and a global prevalence estimate of 28.0 percent for depression during COVID-19 (Nochaiwong et al., 2021). This Canadian sample of social workers falls well above these levels of reported depression, with COVID-19 factors being significant in increased depression. Of further note, we found weak evidence suggesting that those with lower personal income (under $50,000) were twice as likely to be depressed when compared with the other income groups.

One fifth (20.8%) of this sample reported PTSD, which is a troubling number of professionals living with PTSD. This rate is quite comparable to global rates of PTSD during COVID-19 (24.1%) reported in a recent systematic review (Nochaiwong et al., 2021), and over a quarter (26.1%) of social workers met the diagnostic criteria for PTSD in a US study (Holmes et al., 2021). Furthermore, our study indicated each additional COVID-19 adversity was associated with a 29 percent higher risk of having PTSD.

Anxiety was reported in one in every six social workers in this sample (15.7%). As compared with general population pre-pandemic norms in Canada anxiety was reported at 2.6–4.6 percent (Dobson et al., 2020; Gilmour, 2016) and this is substantially higher than the general population. However, this is lower than anxiety reported by health care workers in another study conducted during COVID-19 at 25.8 percent (Salari et al., 2020), and lower than global levels during COVID-19 at 26.9 percent (Nochaiwong et al., 2021). In our sample, there was a statistical trend that social workers with lower personal income (under $50,000 annually) had a higher prevalence of anxiety.

Our study indicated that 25 percent of the social workers in this sample had 3 or more ACEs, and only 10 percent had 4 or more ACEs, which was markedly lower than we had anticipated based on earlier studies of social work students. It is possible that the marked difference in ACEs prevalence could be due to country level differences, since the average levels of interpersonal violence, substance dependence, and parental incarceration are lower in Canada, where the current study was conducted, in comparison to the United States where the earlier studies of students occurred (Gannon, 2001; Ritchie and Roser, 2019; Walmsley, 2013). It could also be the case that the current sample of social workers was notably older than the previous studies’ student populations, and the observed disparity in ACE history may reflect differences by age cohort.

### Limitations

Caution is needed when interpreting the findings due to some limitations of the study. This study used a convenience sample, and therefore, findings cannot be generalized to the Canadian population of social workers. In addition, due to the relatively small number of non-White social workers in our sample, it was necessary to combine all visible minority respondents into one category. We recognize that race and ethnicity, among other aspects of identity, are highly nuanced, and the White/visible minority dichotomization in our study does not capture the full constellation of identities and lived experiences of social workers in Canada. Although we would have preferred to have an oversampling of racialized respondents to conduct racial/ethnic-specific analyses, the prevalence of racialized respondents in our study (14.4%) is actually slightly higher than some other Canadian studies of social workers (e.g. Yan and Chan, 2010, 12%). Finally, the study was conducted in January and February of 2021, before there was widespread access to vaccines in Canada. As of 18 August 2022, 82.4 percent of Canadians have received at least two doses of the COVID-19 vaccination (COVID-19 Tracker Canada, 2022).

It is possible the extremely high levels of COVID-19 related concerns observed in this study may be declining, but additional research is needed to confirm this hypothesis.

## Conclusion

This study focused on Canadian social workers’ adversities, mental health, and resilience during the COVID-19 pandemic. The increased levels of depression, PTSD, and anxiety reported in the survey point to the need for workplaces to develop policies and practices in urgently responding to supporting social workers and their well-being. Social workers’ best chances of being resilient rely on their environment providing the right supports. This is best done on a collective basis, at organization and agency levels, with policies and practices intentionally developed for the current pandemic conditions and mental health impacts on employees. While individual self-care practices are equally as valuable for overall well-being, benefits of these practices are eroded if the workplace is not just as invested in embedding a trauma and resilience informed approach.

As stressed earlier, the process of resilience does not occur in a vacuum for individuals; rather, resilience involves a dynamic interaction between people and their environments that acts to support individuals, families, and communities to overcome adversity (Authors, 2018; Ungar, 2013). Opportunities to create mutual support networks and maintain connections to professional associations to lobby for healthier work conditions are some further strategies to build resilience. Especially vulnerable are social workers early in their professional careers and particularly now, as entry into the workforce is occurring in the context of a pandemic. These and other strategies to sustain the profession into the next and future generations are needed, as this may be a defining moment in the social work field. Now, more than ever, a trauma and resilience informed approach is required in ensuring well-being and addressing serious mental health issues as reported by social workers at alarming rates. Social workers need specialized supports as they deal with COVID-19 conditions, and the subsequent consequences of this pandemic. Enhanced and innovative supports provided by agencies, organizations, and leadership teams are critical in fostering resilience among all social workers during these troubled times.
